# Genetic Diversity in the Capsid Protein-Coding Region of HIV-1 Circulating in Benguela, Angola: Implications for Primary Resistance to the Novel Capsid Inhibitor Lenacapavir

**DOI:** 10.3390/v17050711

**Published:** 2025-05-16

**Authors:** Gonçalo Queirós, Lesya Yefimenko, Filomena M. Pereira, João Piedade

**Affiliations:** 1 Instituto de Higiene e Medicina Tropical, IHMT, Universidade NOVA de Lisboa, UNL, Rua da Junqueira 100, 1349-008 Lisboa, Portugal; goncalopinhoqueiros@gmail.com (G.Q.); a596327@gmail.com (L.Y.); 2 Global Health and Tropical Medicine, GHTM, Associated Laboratory in Translation and Innovation Towards Global Health, LA-REAL, Instituto de Higiene e Medicina Tropical, IHMT, Universidade NOVA de Lisboa, UNL, Rua da Junqueira 100, 1349-008 Lisboa, Portugal; flmpereira@ihmt.unl.pt

**Keywords:** HIV-1, Angola, molecular epidemiology, subtypes, antiretroviral therapy, lenacapavir, drug resistance mutations

## Abstract

In 2023, the HIV-1 pandemic claimed around 630,000 lives worldwide due to AIDS-related complications. Its burden is significantly heavier in Sub-Saharan Africa, where an increased HIV-1 genetic diversity is common, which increases the risk of resistance to antiretroviral (ARV) drugs. This study aims to update the molecular epidemiology of HIV-1 in Angola, focusing specifically on the *gag* gene, which is often overlooked, and to assess the potential viability of lenacapavir (LEN)-based ARV therapy in the region. A total of 243 blood samples were collected from ARV-naïve, HIV-infected patients at the General Hospital of Benguela, city of Benguela, Angola. The capsid-encoding region of HIV-1 proviral DNA was amplified by PCR and sequenced. Phylogenetic analysis was performed using the maximum likelihood method, and genome recombinant forms were characterised through bootscanning analysis. Primary resistance mutations to LEN were identified using Stanford University’s HIVdb algorithm. Among the 80 successfully sequenced samples, 13 different genetic forms/subtypes were identified, with unique recombinant forms (URFs) (37.5%, 30/80) and subtype C (31.25%, 25/80) being the most prevalent. Regarding resistance mutations, none were detected, apart from four polymorphic mutations. These findings reinforce Angola’s position as a transitional HIV-1 hotspot between the genetically highly diverse Central Africa and the subtype C-dominated Southern Africa, while also supporting the potential effectiveness of LEN-based regimens for treatment and prevention of HIV-1 infections in the future.

## 1. Introduction

With approximately 39.9 million people living with HIV (PLWH) and over 630,000 lives lost directly to AIDS-related conditions in 2023 [1], the HIV-1 pandemic is a contemporary health issue that is yet to be resolved. The virus’ ability to cause a chronic infection and a high level of genetic diversity are the foundations behind its global-scale dissemination and the development of resistance against antiretroviral (ARV) drugs. This highlights the precarious situation of Sub-Saharan Africa, which hosts the biggest subtype diversity worldwide relating to its role in the origin of the pandemic [2].

The HIV-1 epidemic in Angola is characterised by a high prevalence of recombinant forms and various non-B subtypes, from which C and F1 emerge as particularly prominent, presenting a relatively diverse genetic profile. However, it is important to note that this subtype distribution is not homogenous across the country [3]. While northern and central regions tend to be more diverse, with more recombinant forms, southern Angola deviates from this trend. Southern regions, including Benguela, tend to have an increased prevalence of subtype C, like the rest of southern Africa, which can make up to 40% of the cases [4]. Concerning Angola’s infection burden, as of 2023, it registered 320,000 PLWH, with almost two-thirds of them being women, and 12,000 people died due to AIDS-related causes [5]. Additionally, access to antiretroviral therapy (ART) was still at 50% for PLHW in Angola, which is notably low considering that 77% of PLWH worldwide have access to ART. Regarding ARV drug resistance, the latest reports from Angola indicated that resistance mutation prevalence to nucleoside reverse transcriptase inhibitors (NRTI), non-nucleoside reverse transcriptase inhibitors (NNRTI), and protease inhibitors (PI) in naïve PLWH can reach up to 40%, 30%, and 8%, respectively [4,6]. This highlights Angola’s high risk for transmitted drug resistance and the need for a new strategy to assess this problem.

In August and December of 2022, the European Medicines Agency (EMA) and the U.S. Food and Drug Administration (USFDA) approved a new pharmaceutical for the treatment of multidrug-resistant HIV infections, lenacapavir (LEN). It is a long-acting drug with an innovative target, the HIV-1 capsid (CA), establishing a new class of ARVs, the capsid inhibitors. By targeting this viral component, LEN binds to the phenylalanine–glycine-binding pocket (FG) and inhibits essential capsid-mediated interactions with cellular co-factors associated with this region. These include nucleoporins, which allow the nuclear import of the pre-integration complex, and RNA processing enzymes that guide the pre-integration complex to transcriptionally active sites [7,8]. Furthermore, it increases capsid stiffness, which is detrimental to proper capsid disassembly in the early stages and capsid maturation in the later stages of the replication cycle [7]. Its multifaceted in vitro effects, in vivo clinical trial results [9,10], and ultra-long-acting nature establish LEN as a new frontier in ART, with the potential to improve adherence and treat multidrug-resistant HIV-1.

The clinical introduction of LEN raised concerns about resistance mutations to the novel inhibitor. Several studies have reported amino acid replacements associated with a reduction in efficacy to LEN, PF-74, or GS-CA1, the last two being structural analogues of LEN. As expected, most documented substitutions are localised in the FG-binding pocket, affecting the compound’s affinity to the cellular co-factors, with further sites counteracting the changes in stiffness induced by LEN [7,11]. Moreover, a direct correlation was observed between the impact of a mutation on viral fitness and the level of resistance it confers [12]. This means that mutations associated with a high-level resistance, also heavily impact the virus’ ability to replicate. According to previous resistance analysis using sequences in online databases, under 1% of sequences had resistance mutations to LEN [13,14]. However, it appears that recent additions to the resistance mutation list may have changed this.

To address the potential of LEN-based therapeutics in Angola and update the molecular epidemiology of the region, specifically targeting the often-overlooked *gag* gene, HIV-1 subtype diversity was characterised, including the genetic structure of unique recombinant forms (URFs) found, along with an automatic and manual LEN resistance mutation assessment.

## 2. Materials and Methods

### 2.1. Sample Collection

As part of a broader cross-sectional study, run between August 2016 and January 2017, patients who were hospitalised, in urgent care, or attending outpatient appointments at the General Hospital of Benguela, in the city of Benguela, Angola, were tested for HIV-1 and HIV-2 using two rapid tests per participant, “HIV 1/2” (Healgen Scientific, Houston, TX, USA) and “Anti-HIV 1/2 Test” (Türklab, İzmir, Turkey). They are both 3rd generation rapid tests which do not separate reactive results to HIV-1 or HIV-2 antibodies (Abs). For patients who tested positive on at least one of the tests, a peripheral blood sample was collected, with an aliquot being stored into Whatman™ Indicating FTA^®^ Elute Micro Cards (Cytiva, Marlborough, MA, USA), while the rest was processed into serum and stored at −20 °C. Both the sera and the FTA^®^ Cards were transported to the Institute of Hygiene and Tropical Medicine (IHMT), NOVA University Lisbon, where the serum was re-tested using the rapid test “Hexagon HIV” (Human Diagnostics, Wiesbaden, Germany), to differentiate between Abs reactive to HIV-1 or HIV-2. In the end, this study included 243 dried blood samples.

General sociodemographic and basic epidemiological data were also collected through a voluntary survey completed by patients who signed the informed consent form.

### 2.2. FTA^®^ Card Disc Preparation

To prime the proviral DNA in the FTA^®^ Elute Micro Cards (Cytiva, Wilmington, DE, USA), a purification protocol was followed according to the manufacturer’s instructions, with in-house minor modifications, using QIAcard™ FTA^®^ Wash Buffer (QIAGEN N.V., Hilden, Germany).

### 2.3. Nested PCR and Sequencing of the Capsid-Encoding Region

Two consecutive nested PCR protocols were used to amplify the capsid-encoding region of HIV-1 proviral DNA. In both protocols, the Supreme NZYTaq II 2× Green Master Mix (NZYtech, Lisbon, Portugal) was utilised. After selecting the amplicons with the expected size via 1.2% agarose gel electrophoresis, each PCR product was sent to STAB VIDA (Caparica, Portugal) for Sanger nucleic acid sequencing, covering both amplicon strands.

The sequences for the set of primers used in the first protocol (Table 1) were obtained from T. Saint-Joannis and D. Descamps (personal communication), from the ANRS-MIE (*Agence Nationale de Recherche sur le SIDA et les Hépatites Virales—Maladies Infectieuses Emergentes*, France) resistance study group. After testing various annealing temperatures and primer combinations, a three-step nested PCR protocol was established. The first reaction used primers CA_S1 and CA_AS1, the second used CA_S1 and CA_AS2, and the third used CA_S2 and CA_AS2. Annealing temperatures were set at 56 °C, 60 °C, and 55 °C for the first, second, and third reactions, respectively, yielding an 801-bp amplicon.

For the second protocol, two primer sequences were obtained from [15]. Modifications were made to the forward primer, 3MA-F, to better account for the anticipated genetic diversity of the samples, creating the 3MA-F2 primer (Table 2). The second protocol involved a regular nested PCR using 3MA-F2 plus CA_AS1 as outer primers for the first reaction, and CA_S1 plus 5NC-R as inner primers for the second reaction, with an annealing temperatures at 52 °C and 55 °C, respectively, amplifying a 951-bp DNA fragment.

### 2.4. Sequence Editing and Analysis

The sequence name format was set as AO, the alpha-2 code for Angola, followed by the respective sample number. The sequences were edited using the BioEdit Sequence Alignment Editor [16], version 7.0.9.0. and then submitted to the Basic Local Alignment Search Tool (BLAST) program, version 2.16.0 (NIH, Bethesda, MD, USA) [17], to confirm their identity as HIV-1 *gag*. A multiple sequence alignment was created through MAFFT [18], version 7, featuring the study sequences and several reference sequences of known pure subtypes and circulating recombinant forms (CRFs), obtained from GenBank [19] and the Los Alamos HIV sequence database (https://www.hiv.lanl.gov/, accessed on 2 May 2024). These sequences were selected based on geographical origin and similarity to study sequences. The multiple alignment was then submitted to the Gblocks program, version 0.91b, to cleanse any misaligned or divergent positions [20]. Maximum likelihood trees were constructed, with branch support assessed with bootstrapping analysis set at 1000 iterations, using the IQ-TREE web server program [21]. The most appropriate nucleotide substitution model was defined by IQ-TREE as being the Transition model (TIM). Considering the alignment, the two equal transversion rates were the following: A<->C = G<->C and A<->T = G<->T. The constructed trees were viewed and edited using the FigTree software (http://tree.bio.ed.ac.uk/software/figtree/, accessed on 27 May 2024), version 1.4.4. When indicated, study sequences were subjected to recombination analysis by bootscanning on SimPlot [22], version 3.5.1, using the neighbour-joining model with 1000 bootstrap iterations, a window of 120 to 200 nts. and steps of 20 nts. They were also assessed for APOBEC-induced mutations through the Stanford University HIVdb program [23], version 9.8, which was ultimately used to evaluate the presence of resistance mutations to LEN. In addition, mutations listed in the ANRS-MIE and IAS-USA databases were manually searched, as well as those described in the literature as reducing susceptibility to LEN or its analogues (Table 3).

HIV-1 sequences obtained in this study were submitted to the GenBank database under the accession numbers PQ513442 to PQ513518 (Table S1).

## 3. Results

### 3.1. Sociodemographic and Epidemiological Analysis of the Sample Group

The basic sociodemographic and epidemiological data of the 243 patients included in this study are summarised in Table 4. The youngest person enrolled was 5 days old, while the oldest was 80 years old, with more than half identifying as female (54%, 131/243). The majority were either single (36%, 87/243) or married (35%, 84/243), and most had some level of education (65%, 157/243). They came from several distinct municipalities within the province of Benguela, with the largest group (38%, 92/243) from the city of Benguela itself. At the time of the HIV diagnosis, 16% (39/243) of patients were co-infected with either syphilis (9%, 21/243), hepatitis B virus (HBV) (7%, 16/243), or both (1%, 2/243). In total, 12% (30/243) of them reported a previous sexually transmitted infection (STI).

### 3.2. Phylogenetic Characterisation of the Sequences

After purification of all 243 sample discs from FTA^®^ cards, 180 were tested using the first PCR protocol, yielding 38 positive results. Of these, 24 samples produced usable sequencing products, excluding those with very noisy chromatograms or truncated sequences. The remaining 219 sample discs, excluding those that had already produced usable sequences, were re-purified, and the second PCR protocol was applied. This resulted in 60 positive samples, 56 of which yielded successful sequence products. Overall, 80 high-quality sequences were obtained across both protocols, representing approximately one-third of the total sample set (32.9%, 80/243), with sequence lengths ranging from 699 to 923 bp.

**Figure 1:**
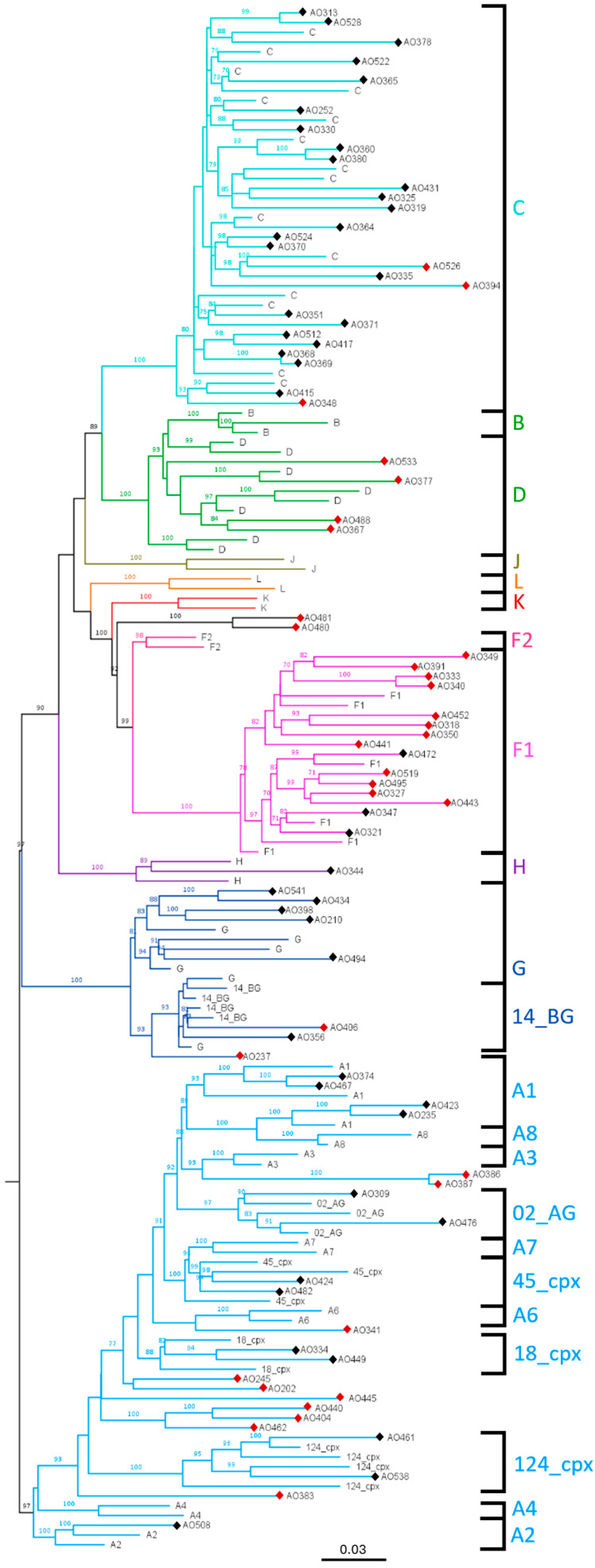
Maximum likelihood phylogenetic tree comprising 158 capsid-encoding sequences, with subtype clusters highlighted in different colours. Black diamonds (◆) indicate study sequences that were successfully classified, while red diamonds (◆) represent unclassified study sequences selected for recombination analysis. The tree was midpoint-rooted, and bootstrap values below 70% were omitted to enhance visual clarity.

As previously stated, reference subtype sequences, including CRFs, were retrieved from the Los Alamos HIV sequence database to be used in phylogenetic tree building. Additionally, subtyped sequences from GenBank were acquired to accommodate study sequences with divergent identities, mostly based on BLAST hits with study sequences. In total, a 79-reference sequence dataset was used in the final tree, which together with the study sequences, comprised 159 sequences. They were aligned and cleaned, resulting in a 636 bp-long alignment used to construct the maximum likelihood phylogenetic tree of Figure 1. One of the study sequences, AO470, was not included in the final tree due to its significant negative effect on tree topology.

**Figure 2:**
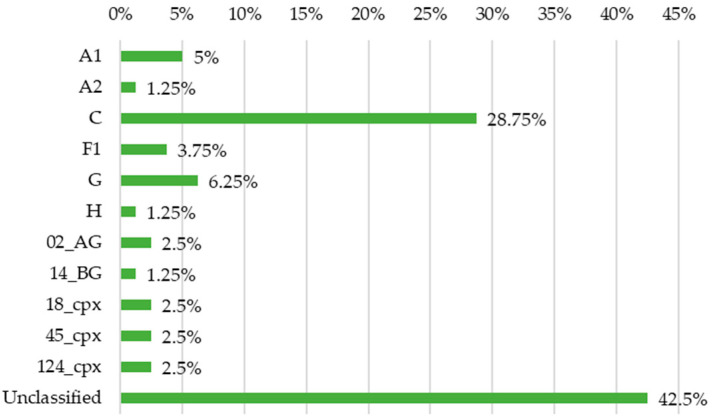
Distribution of HIV-1 subtypes and recombinant forms based on tree interpretation.

Most pure subtype sequences clustered into monophyletic groups, supported by bootstrap values above 70%, except for A1, which appeared to split into two separate clades, and B and D subtypes, which segregate together due to their genetic proximity. Regarding the CRFs, they clustered according to their genetic mosaic structures: CRFs 02_AG, 18_cpx, and 45_cpx aligned with subtype A in the capsid-encoding region; CRF14_BG aligned with subtype G; and CRF124_cpx displayed an A-to-G recombinant breakpoint within the same region. Subtype classifications were summarised in Figure 2. The “Unclassified” category was assigned to sequences exhibiting high genetic divergence or positional ambiguity. This group included sequences that formed relatively long branches, segregated in isolation, or clustered into monophyletic groups exclusively with other study sequences.

Each study sequence was submitted to the Stanford University HIVdb for screening of APOBEC-induced mutations, with a total of eight sequences exhibiting such mutations (Table S2). Sequence AO394 was particularly hypermutated, with 18 mutations, many resulting in stop codons. This explains the sequence’s extended branch in the phylogenetic tree, which was the primary reason it was initially considered unclassified. However, due to its central positioning within the phylogenetically consistent C cluster, segregating among reference sequences, it was later classified as a pure subtype C, with its genetic distance attributed to the high number of APOBEC-induced mutations. All these sequences were subsequently excluded from LEN resistance mutation analysis.

Unclassified sequences, excluding AO394 (*n* = 33), were analysed for recombination through bootscanning, utilising the same dataset as the reference sequences for the tree (Figure S1). Sequence regions where bootscanning analysis did not produce a consensus were classified as undetermined (U). Based on the recombination analysis, nearly all the previously unclassified sequences were identified as recombinants (Table S3), with the exceptions of AO341, which appears to belong to sub-subtype A6; AO406, which is likely a CRF14_BG or a G subtype related to the CRF origin; and AO526, which is a divergent C subtype.

**Figure 3:**
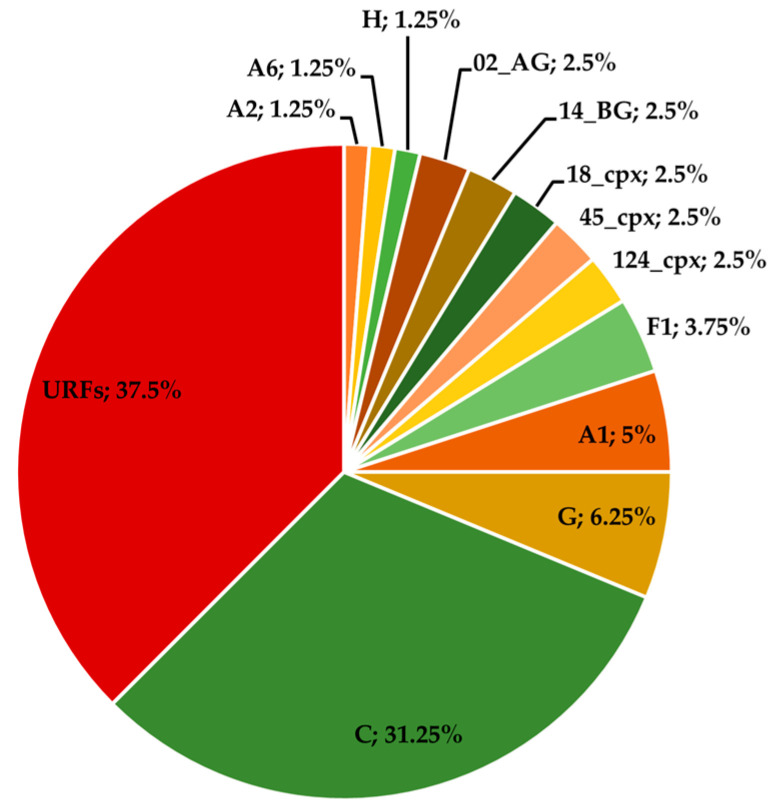
Distribution of HIV-1 subtypes and recombinant forms based on phylogenetic analysis, including recombination analysis using SimPlot.

Based on the conclusions from the SimPlot analysis, the subtype proportions were updated, with the addition of one A6 sequence (AO341), two C sequences (AO394 and AO526), and one CRF14_BG sequence (AO406). All sequences in Table S3 were classified as URFs, and the final results are presented in Figure 3.

### 3.3. Resistance Analysis to LEN

Resistance analysis was initially performed on 72 sequences, excluding those with APOBEC-induced damage, using the HIVdb program and the IAS-USA and ANRS-MIE reports. No official mutations associated with LEN resistance were found. However, two distinct polymorphic mutations were detected at position 107, a site associated with drug resistance, in seven different sequences. Mutation T107A was found in sequence AO350 and mutation T107S was found in sequences AO333, AO340, AO344, AO383, AO406 and AO431. They were stated as “polymorphic mutations that have been reported in combination with other LEN-associated mutations” with unknown effects on drug susceptibility.

In addition to the previously mentioned polymorphic mutations, mutations associated with reduced susceptibility to LEN, PF-74, or GS-CA1 were identified based on a literature review. S41A, reported in [24] as affecting susceptibility to PF-74 by compensating for a loss of viral fitness, was found in two sequences, AO344 and AO349. Another mutation, T200I, found in sequence AO365, was associated with increased capsid stability, negatively affecting susceptibility to LEN [26]. It is noteworthy that, overall, sequence AO344 exhibited two distinct polymorphic mutations: S41A and T107S.

## 4. Discussion

Here, we analysed both the subtype distribution of the capsid-encoding region and the presence of resistance mutations to the newly approved LEN in HIV-1 strains circulating in the province of Benguela, Angola. To achieve this, two amplification protocols were set up. The first was prematurely discontinued due to its negative selection of subtype C sequences. This issue became evident through an initial rapid subtyping using the REGA subtyping tool [27], which revealed that only one out of the 24 sequences obtained from the first protocol belonged to subtype C, an outcome highly inconsistent with previous subtype distribution analyses in the region [4]. The underlying cause stemmed from a primer mismatch with subtype C sequences. Consequently, a new primer pair was considered, and a second amplification protocol was implemented. Although this resolved the issue of the negative selection of the C subtype, sequences were produced in only one-third of the samples (80/243). Several factors can influence the rate of amplification of proviral DNA. Given that the samples were collected from late 2016 to early 2017, their age may have compromised the quality of the DNA preserved within the cellulose matrix of the cards. Additionally, exposure to humidity may have further contributed to degradation of the DNA template. Taken together, particularly the age of the samples, these factors could impact the generalizability of our findings, as they may not fully represent current resistance patterns and relative proportions of circulating subtypes in the area, giving the rapid evolution of the virus. Moreover, viral loads were not measured at the time of sample collection, introducing uncertainty, as variations in viral load could influence the amount of proviral DNA present in the sample, potentially affecting the amplification results [28]. Finally, as experienced in this study, HIV-1 genetic diversity poses a significant challenge to successful amplification, particularly since many research tools and protocols are optimized for subtype B, which is most common in high-income countries, thereby contributing to this bias.

Regarding the results of the phylogenetic analysis (Figure 1), tree topology was consistent, with different subtypes forming individual clades supported by significant bootstrap values of over 70%. It is worth noting, however, that sequences from sub-subtype A1 did not cluster into a single monophyletic group but instead formed two distinct lineages within the larger A clade. This is documented in other articles that assembled trees using subtype A sequences [29]. It suggests that the reference sequences may be misclassified, the genetic distance between A sub-subtypes may not be uniform across the entire genome—particularly considering that the *gag* region is more conserved—or they may represent previously undocumented A sub-subtypes.

Based on tree interpretation, there is a great diversity of subtypes circulating in Benguela, with capsid sequences belonging to pure subtypes A1, A2, C, F1, G, and H. Even though some sequences segregated with CRF02_AG, CRF14_BG, CRF18_cpx, CRF45_cpx, and CRF124_cpx, we cannot necessarily infer that these are all recombinant sequences. All mentioned CRFs, except for CRF124_cpx, correspond to a pure subtype across the capsid region, with no recombination breakpoints. This suggests that these sequences may simply represent the pure subtype lineage from which the respective CRFs originated. CRF02_AG is a pure A subtype, CRF14_BG is a pure G subtype, CRF18_cpx is a pure A1 subtype, and CRF45_cpx is a pure A subtype in the studied genome region. This is particularly evident for CRF14_BG, as some reference G subtype sequences originating from Portugal and Spain, where this recombinant form was initially described, segregate in the cluster. This may reflect the historical link between Portugal and its former African colonies. It has been recorded that the G subtype lineage that originated this CRF was circulating in Angola, and its introduction into Portugal was probably during the establishment of Angola’s independence [30]. Based on this, the study sequences that clustered within the CRF14_BG clade most likely belong to that ancestral subtype G lineage. CRF124_cpx, on the other hand, has a recombination crossover from A-to-G within the capsid region, specifically at nucleotide 1371, which strengthens the claim that the study sequences that cluster within this CRF, showing high bootstrap support and containing the same recombination breakpoint, belong to CRF124_cpx.

Regarding subtype distribution based only on tree interpretation, C was by far the most common pure subtype at 28.75% (23/80), with all the other pure subtypes and CRFs coming well behind, representing between 1.25% (1/80) and 6.25% (5/80) each. The remaining sequences, specifically 42.5% (34/80), could not be directly classified through phylogenetic tree analysis. Characteristics such as long branch lengths, isolated positioning, or the formation of distinct monophyletic groups composed exclusively of study sequences indicated potential genetic divergence. Consequently, most sequences exhibiting at least one of these features were not immediately classified and were instead selected for recombination analysis by bootscanning. It was also important to rule out the presence of APOBEC-induced mutations in the study sequence, as they can generate genetic divergence that is not epidemiologically or clinically significant. Eight sequences were found to harbour these mutations (Table S2), with AO394 exhibiting extensive hypermutation, with 18 distinct mutations. This explains why AO394 is supported by a long branch, which, given its positioning in the tree, can help define it as a hypermutated pure C subtype. All of these sequences were excluded from the resistance mutation analysis, as the presence of APOBEC-mediated hyperadenylation suggests that they may originate from replication-incompetent viruses, making them clinically irrelevant and unlikely to contribute to disease progression.

The remaining unclassified sequences were subjected to recombination analysis, using the same reference sequences from the phylogenetic tree dataset. This was refined to include more sequences from geographically closer countries, which is expected to result in the selection of genetically closer sequences, making it more suited for recombination analysis. Smaller steps, mostly 20 nt., were prioritised as they can give us more consistent breakpoints [31] and although smaller windows are not ideal in full genome analysis, window sizes between 120 and 200 nts. were used because the analysed fragment was under 650 bp. Bootscanning was performed at 1000 bootstrap iterations to improve support and accuracy and values under 70% were considered non-supportive. Using these parameters, bootscanning analysis of AO341, AO406, and AO526 revealed that these sequences are not recombinants, but rather belong to established subtypes. Specifically, AO341 appears to be a divergent A6 subtype, AO406 aligns with either a CRF14_BG or a genetically related G subtype, and AO526 is classified as a C subtype. The remaining sequences exhibited at least one breakpoint, with their mosaic structure justifying their positioning on the phylogenetic tree (e.g., AO391, which is mostly F1, clustered in the F1 clade). AO470, initially excluded from the final tree due to its impact on the phylogenetic relations between subtype A2 and the subtype G cluster, is a complex URF involving A2, F2, and G (Table S3), which accounts for this observation. As anticipated, sequences that formed monophyletic groups with high bootstrap support showed similar patterns of recombination, including closely localised breakpoints and comparable bootscanning graphs when using the same background sequences for analysis. This is the case for the sequences grouped in Table S3, and for some of these groups, it may suggest the potential emergence of new CRFs. One example is the group comprising sequences AO404, AO440, and AO462, which share the same A1/G recombination pattern and were obtained from patients from different municipalities in the province of Benguela, showing a seemingly low probability of an epidemiological link. AO404 originated from a widowed 59-year-old female from Chongoroi, AO440 from a married 52-year-old female from Cubal, and AO462 from a 14-year-old male from Ganda. Further analyses would have to demonstrate a similar full-genome mosaic structure between the three viruses and confirm that the infections were epidemiologically unrelated, as these are the criteria for establishing a new CRF.

It has been documented that recombination throughout the HIV-1 genome is inconsistent, with certain regions being more or less prone to recombination, referred to as hot and cold spots, respectively. In the SimPlot analysis conducted in this study, 35 different crossovers were identified. Using the breakpoints referenced in [32], 30 of these crossovers were localised in hot spots with significant *p*-values, two in cold spots with significant *p*-values, and three in unsupported cold spots. These findings substantiate the presence and validity of these crossovers. Given the geographical context of Angola, associated with a viral high genetic diversity and a high prevalence of new recombinant forms, an increased frequency of URFs is expected.

After considering both the HIVdb and SimPlot results, the final subtype proportions are presented in Figure 3. When compared with the initial tree results (Figure 2), subtype diversity shows a slight increase. The C subtype reaffirmed its position as the most common subtype at 31.25% (25/80), second only to the URF category, which accounted for 37.5% (30/80). Most of the CRFs found were either described for the first time in Angola or reported circulating in Central Africa. Specifically, CRF124_cpx, which was initially described using sequences from Angola [33], was not surprising to find. This distribution reflects Benguela’s role as a buffer zone between the more diverse Central Africa and the predominantly subtype C region of Southern Africa.

The next step involved assessing primary resistance to LEN based on the presence of resistance mutations in the capsid region. No resistance mutations were detected by the HIVdb program or through manual analysis using IAS-USA and ANRS-MIE reports, but other mutations in known resistance sites were observed. T107A and T107S, localised at the same site near the FG-binding pocket [7], are polymorphic mutations selected by LEN but do not contribute to its resistance. These mutations were reported in the CAPELLA clinical trial, which tested the efficacy of a LEN-based regimen in heavily treatment-experienced individuals with documented resistance to other ARV classes [11]. Initially, T107A appeared to confer a low resistance level, being associated with a penalty score of 20 in HIVdb, but further analysis ruled it out [34]. The fact that seven sequences presented mutations at position 107 is not unprecedented, as this position is one of the least conserved resistance mutation sites [13,35]. While these findings suggest an absence of primary resistance to LEN, concerns remain due to the association of these polymorphisms with the development of overt resistance. These results align with previous reports on LEN resistance, with Nka et al. [13] and Troyano-Hernáez et al. [14] reporting resistance rates of under 1%, despite having a considerably larger sequence pool, but a more limited list of resistance mutations compared to the present study, even when only considering Stanford University’s.

In addition to using the HIVdb program, and the IAS-USA and ANRS-MIE compilations to identify LEN resistance mutations, a manual analysis was performed to search for additional mutations described in the literature. LEN is a recently introduced pharmaceutical, and research on LEN resistance is still very limited and rapidly evolving. As highlighted by the previous mention of LEN resistance mutation prevalence, it is crucial to keep this information updated. The first potential resistance mutation, found in two sequences, was S41A described by Zhou et al. [24], as selected by and reducing susceptibility to PF-74. S41A is not located in the FG-binding pocket, suggesting that its effects are due to partially restoring viral fitness loss from other resistance mutations, rather than directly impacting the affinity to the drug. Even though the assay was not performed using LEN, PF-74 is a structural analogue with similar effects on viral replication, binds to the same pocket in the viral capsid, and selects for similar resistance mutations, like Q67H. The second potential mutation, T200I, found in one sequence, slightly reduces susceptibility to LEN [26], with a more pronounced effect when counteracting mutations that increase susceptibility to it. This is thought to be due to a stabilising effect on the viral capsid, suggesting that T200I may function as a compensatory mutation, enhancing viral fitness after acquisition of a destabilising resistance mutation in the capsid. Since both mutations are not officially recognised, they were not considered when determining the rate of sequences with primary resistance.

Several limitations should be considered regarding the techniques and outcomes of this study. Firstly, the use of proviral DNA in molecular epidemiology analysis can result in the detection of archived or replication-incompetent viruses, particularly in treatment-naïve individuals. The latter are not necessarily clinically relevant and archived viruses may harbour resistance mutations of dubious impact on long-term therapy. Considering this, the drug resistance findings must be interpreted prudently in terms of their direct impact on ART. Secondly, as mentioned before, there was a relatively low success rate of the PCR reactions, due to factors like viral load, genetic diversity, and sample quality, resulting in a moderate study sample size of 80 sequences. Additionally, the Sanger DNA sequencing technique fails to detect minor viral populations present in the individual quasispecies due to its limited detection threshold, potentially overlooking less fit, drug-resistant viral strains. Finally, it is important to emphasise that the subtype analysis was limited to the capsid-encoding region, meaning the complete viral subtype could not be determined.

## 5. Conclusions

This study addressed the molecular epidemiology of HIV-1 in Angola and provided valuable insights into LEN primary resistance. It established that subtype C (31.25%, 25/80) is by far the most common pure subtype circulating in the province of Benguela, with a great diversity of other pure subtypes also represented (A1, A2, A6, F1, G, and H). Recombinant forms potentially account for half of the circulating viruses, including CRF02_AG, CRF14_BG, CRF18_cpx, CRF45_cpx, and CRF120_cpx (2.5%, 2/80, each), and the most significant prevalence of apparent URFs (37.5%, 30/80), which is to be expected given the region’s viral genetic diversity reported in previous reports [3]. Overall, these findings position the province of Benguela as a transition zone between the recombinant-dominated, highly diverse, Central Africa and the C subtype bastion of Southern Africa [36]. This has direct implications for ART strategies in Angola. The high genetic diversity and recombinant prevalence reinforce the need for tailored, adaptable ART regimens and ongoing genomic surveillance of resistance patterns. Regarding primary resistance to LEN, no official resistance mutation was found. These results offer a glimpse of hope for Angola, which still faces significant challenges in effectively controlling the HIV epidemic. A long-acting regimen like LEN would certainly benefit the country by addressing adherence issues that contribute to therapy resistance and providing a valuable alternative for individuals with multidrug resistance to first-line ARV classes. Ultimately, strengthening molecular surveillance systems locally could further guide public health responses and optimize future ART programs in Angola.

## Figures and Tables

**Table 1:** Primers used in the first PCR amplification protocol.

	Sequence 5′–3′	Location (in HXB2)	CA-Encoding Region (in HXB2)
Outer primers	CA_S1 GAS ATA AAA GAC ACC AAG GAA GC	1066–1088	1186–1879
	CA_AS1 CAT TTC CAA CAG CCC TTT TTC C	2015–2036	
Inner primers	CA_S2 GCA GCA GCT GMC ACA GG	1141–1157	
	CA_AS2 CCT AAA ATT GCY TCT CTG CAT C	1920–1941	

**Table 2:** Primers used in the second PCR amplification protocol.

Sequence 5′–3′	Location (in HXB2)	CA-Encoding Region (in HXB2)
3MA-F2 CAG TAG CAA YCC TCT ATT GTG TRC	1031–1054	1186–1879
5NC-R CCT AGG GGC CCT GCA ATT T	1998–2016	

**Table 3:** Compilation of resistance mutations to LEN organised by source.

LEN Resistance Mutations (Validated)			Polymorphisms of Unknown Effect (Stanford HIVdb)	Literature-Reported Mutations
Stanford HIVdb	IAS-USA	ANRS-MIE		
L56I	L56I	L56I	T107A/C/S	S41A [24]
N57S	M66I	M66I		E45A [7]
M66I	Q67H	Q67H/K/N		Q67A [25]
Q67H/K/N/Y	K70H/N/R/S	K70H/N/R/S		L172I [24]
K70H/N/R/S	N74D/S	N74D/H/K/S		E180A [7]
N74D/K/S	A105T	A105S/T		T200I [26]
A105E/S/T	T107N	T107C/N		
T107C/N				

**Table 4:** Sociodemographic and epidemiological characteristics of the patients included in this study.

Characteristics	
Age (years)	
Mean (Min–Max)	39 (0–80)
Sex (%)	
Female	54
Male	39
Unspecified	7
Marital status (%)	
Single	36
Married	35
Divorced	12
Widowed	10
Unspecified	7
Education level (%)	
No education	28
Primary education	31
Secondary education	28
Higher education	6
Unspecified	7
Previous STIs (%)	
Yes	12
No	42
Does not know/Did not answer	46
Present STI co-infection (%)	
Syphilis	9
HBV	7
Syphilis + HBV	1

## Data Availability

The HIV-1 capsid sequence data generated in this study have been deposited in GenBank, with the corresponding accession numbers listed in Supplementary Materials (Table S1). The original contributions presented in this study are included in the article/Supplementary Material. Further inquiries can be directed to the corresponding author.

## Supplementary Materials

The following supporting information can be downloaded at: https://www.mdpi.com/article/10.3390/v17050711/s1, Table S1: GenBank accession number and subtype classification for each sequence analysed. Table S2: APOBEC-induced mutations identified through HIVdb analysis. Figure S1: SimPlot bootscanning graphs of the sequences submitted to recombination analysis. The red line indicates the 70% threshold. Sequences with related recombination patterns are represented by a single representative sample. Table S3: Recombination patterns and nucleotide breakpoints identified through SimPlot analysis.
