# Supplementary material for: Genetic Diversity in the Capsid Protein-Coding Region of HIV-1 Circulating in Benguela, Angola: Implications for Primary Resistance to the Novel Capsid Inhibitor Lenacapavir
Source: Viruses. 2025 May 16;17(5):711. doi: 10.3390/v17050711 (PMC12116093; doi:10.3390/v17050711)

Article

Genetic diversity in the capsid protein-coding region of HIV-1 circulating in Benguela, Angola: implications for primary resistance to the novel capsid inhibitor lenacapavir

Gonçalo Queirós [1], Lesya Yefimenko [1], Filomena M. Pereira [2] and João Piedade [2,] *

1 Instituto de Higiene e Medicina Tropical, IHMT, Universidade NOVA de Lisboa, UNL, Rua da Junqueira 100, 1349-008 Lisboa, Portugal;
2 Global Health and Tropical Medicine, GHTM, Associated Laboratory in Translation and Innovation Towards Global Health, LA-REAL, Instituto de Higiene e Medicina Tropical, IHMT, Universidade NOVA de Lisboa, UNL, Rua da Junqueira 100, 1349-008 Lisboa, Portugal;
goncalopinhoqueiros@gmail.com (G.Q.); a596327@gmail.com (L.Y.); flmpereira@ihmt.unl.pt (F.M.P.)
* Correspondence: jp@ihmt.unl.pt (J.P.)

Supplementary Materials:

Table S1: GenBank accession number and subtype classification for each sequence analysed.

Study Sequence	Accession number	Subtype	Study Sequence	Accession number	Subtype
AO202	PQ513442	A1/G/A1	AO387	PQ513460	A3/A7
AO210	PQ513516	G	AO391	PQ513500	F1/D
AO235	PQ513451	A1	AO394	-[1]	C
AO237	PQ513463	G/K/G	AO398	PQ513464	G
AO245	PQ513443	A1/H/A1	AO404	PQ513448	A1/G
AO252	PQ513472	C	AO406	PQ513468	CRF14_BG
AO309	PQ513459	CRF02_AG	AO415	PQ513483	C
AO313	PQ513480	C	AO417	PQ513475	C
AO318	PQ513498	F1/G	AO423	PQ513452	A1
AO319	PQ513493	C	AO424	PQ513446	CRF45_cpx
AO321	PQ513502	F1	AO431	PQ513492	C
AO325	PQ513491	C	AO434	PQ513465	G
AO327	PQ513504	F1/G	AO440	PQ513449	A1/G
AO330	PQ513484	C	AO441	PQ513501	F1/D
AO333	PQ513509	F1/H	AO443	PQ513508	F1/G
AO334	PQ513444	CRF18_cpx	AO445	PQ513462	U/A1/G
AO335	PQ513490	C	AO449	PQ513445	CRF18_cpx
AO340	PQ513510	F1/H	AO452	PQ513512	F1/G
AO341	PQ513453	A6	AO461	PQ513470	CRF124_cpx
AO344	PQ513517	H	AO462	PQ513456	A1/G
AO347	PQ513507	F1	AO467	PQ513455	A1
AO348	PQ513482	C/F2/C	AO470	PQ513458	A2/G/F2/A2
AO349	PQ513499	F1/J	AO472	PQ513503	F1
AO350	PQ513511	F1/G/F1	AO476	PQ513450	CRF02_AG
AO351	PQ513488	C	AO480	PQ513513	F2/L/F2/L

AO356	PQ513467	CRF14_BG	AO481	PQ513514	F2/L/F2/L
AO360	PQ513485	C	AO482	PQ513447	CRF45_cpx
AO364	PQ513477	C	AO488	PQ513496	U/B/D
AO365	PQ513487	C	AO494	PQ513469	G
AO367	PQ513495	G/D	AO495	PQ513506	F1/G
AO368	PQ513478	C	AO508	PQ513457	A2
AO369	PQ513479	C	AO512	PQ513476	C
AO370	PQ513473	C	AO519	PQ513505	F1/G
AO371	PQ513489	C	AO522	-[1]	C
AO374	PQ513454	A1	AO524	PQ513474	C
AO377	PQ513497	D/F2/D	AO526	PQ513494	C
AO378	-[1]	C	AO528	PQ513481	C
AO380	PQ513486	C	AO533	PQ513515	C/D/B
AO383	PQ513461	A8/G/A8	AO538	PQ513471	CRF124_cpx
AO386	PQ513518	A3/A7	AO541	PQ513466	G

[1]Three sequences were not submitted to GenBank due to the presence of APOBEC-derived stop codons.

Table S2. APOBEC-induced mutations identified through HIVdb analysis.

Study sequence	*APOBEC mutations*
AO237	G116R
AO252	G101R
AO356	G116R
AO378	E75K, E159K, G223R, R229K
AO394	E75K, E76K, G106E, G127E, E128K, G137K, R143K, E159K, D166N, R173K, E175K, G206K, E213K, M214I, G220E, G222K, G223R, R229K
AO470	G116R
AO522	G60E, G89R, G101R, G137R
AO541	E213K

Table S3. Recombination patterns and nucleotide breakpoints identified through SimPlot analysis.

Study sequences	*Recombination pattern*	*Nucleotide breakpoints (in HXB2)*
AO202	A1/G/A1	1455/1541
AO237	G/K/G	1604/1676
AO245	A1/H/A1	1473/1628
AO318, AO452	F1/G	1623-1646[1]
AO327, AO443, AO495, AO519	F1/G	1613-1620[1]
AO333, AO340	F1/H	1618
AO348	C/F2/C	1475/1635
AO349	F1/J	1623
AO350	F1/G/F1	1293/1463
AO367	G/D	1334
AO377	D/F2/D	1520/1621
AO383	A8/G/A8	1423/1492
AO386, AO387	A3/A7	1529
AO391	F1/D	1605
AO404, AO440, AO462	A1/G	1650-1682[1]
AO441	F1/D	1657
AO445	U/A1/G	1310/1599
AO470	A2/G/F2/A2	1360/1518/1715
AO480, AO481	F2/L/F2/L	1310-1328/1460-1473/1668[1]
AO488	U/B/D	1442/1612
AO533	C/D/B	1433/1623

[1]Some breakpoints exhibit nucleotide ranges due to slight variations in breakpoints across different sequences of the same recombinant type.

Figure S1: SimPlot bootscanning graphs of the sequences submitted to recombination analysis. The red line indicates the 70% threshold. Sequences with related recombination patterns are represented by a single representative sample.

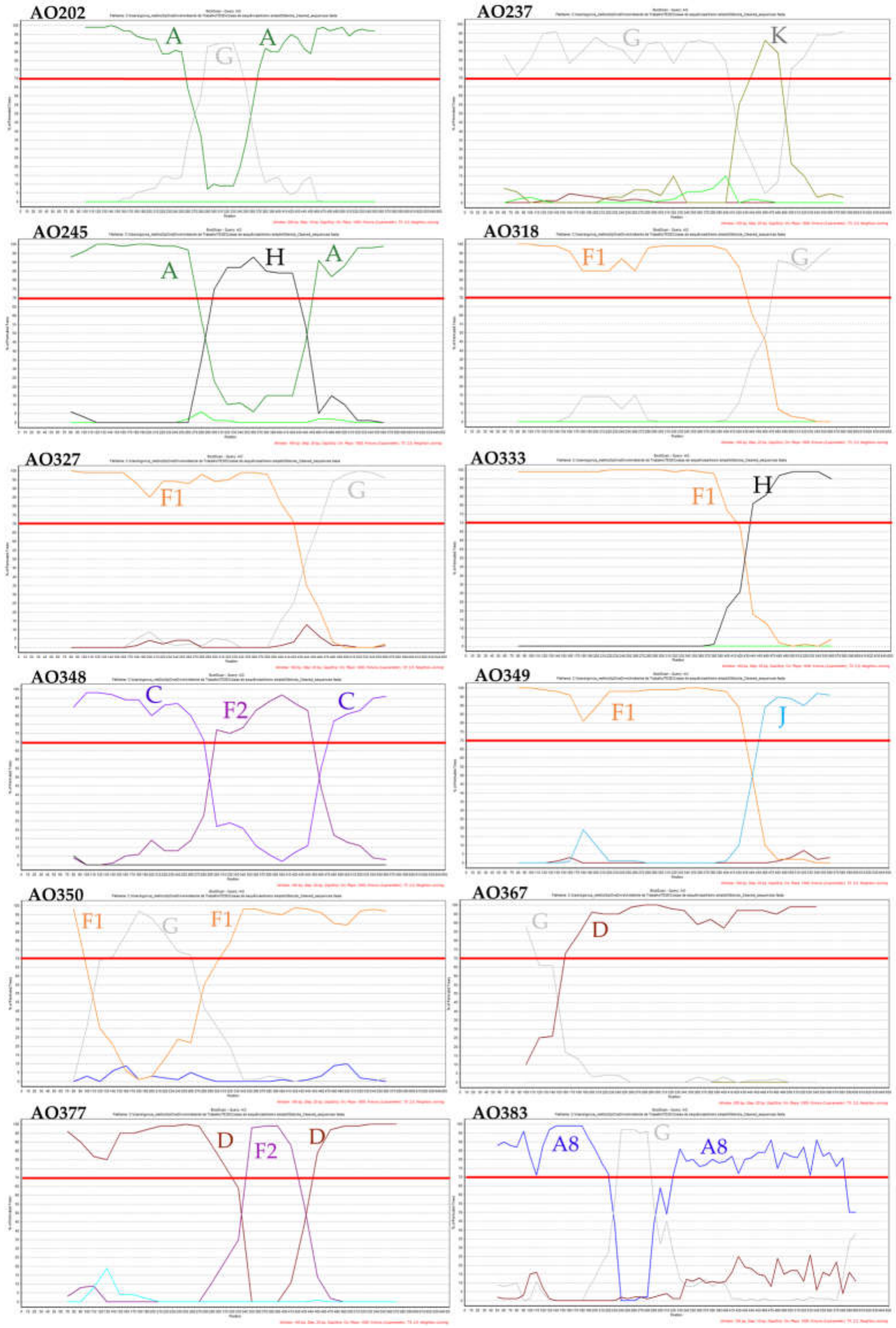

Figure S1: *Cont.*

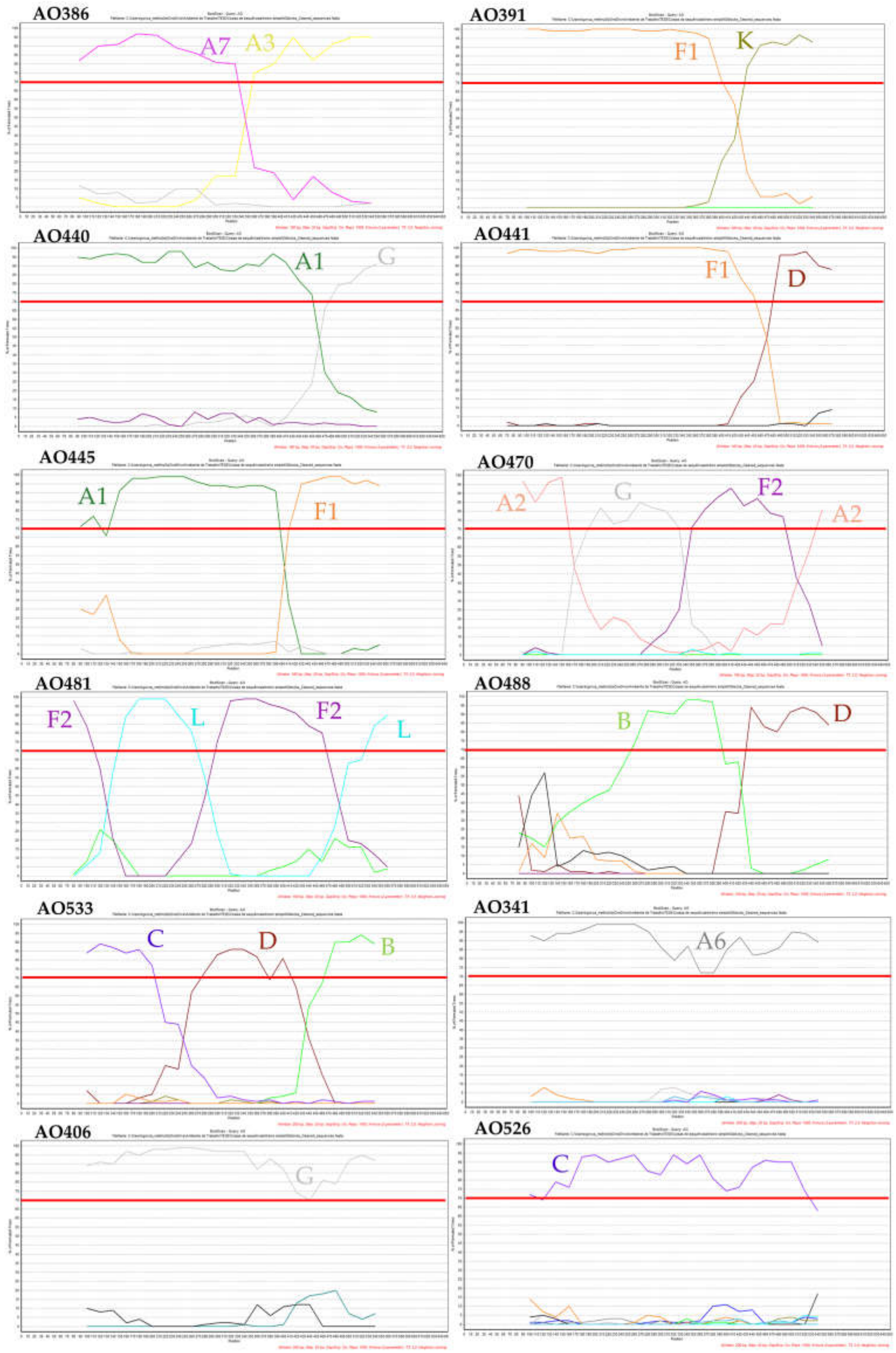

Supplement: Supplementary file 1 [file viruses-17-00711-s001.zip › viruses-3610865-supplementary.pdf]
